# Association of novel lipid indicators with the risk of stroke among participants in Central China: a population-based prospective study

**DOI:** 10.3389/fendo.2023.1266552

**Published:** 2023-10-02

**Authors:** Qin Huang, Li Yin, Zeyu Liu, Minping Wei, Jie Feng, Qing Huang, Yunhai Liu, Zunjing Liu, Jian Xia

**Affiliations:** ^1^ Department of Neurology, Xiangya Hospital, Central South University, Changsha, Hunan, China; ^2^ Department of Neurology, Peking University People’s Hospital, Beijing, China; ^3^ Departement of Chronic Disease, Hunan Provincial Center for Disease Control and Prevention, Changsha, China; ^4^ National Clinical Research Center for Geriatric Disorders, Xiangya Hospital, Central South University, Changsha, China; ^5^ Clinical Research Center for Cerebrovascular Disease of Hunan Province, Central South University, Changsha, China

**Keywords:** triglyceride glucose index, triglyceridemic-waist phenotypes, stroke, metabolic syndrome, visceral adiposity dysfunction

## Abstract

**Background:**

Several easily and inexpensively measured indicators of visceral adiposity dysfunction are currently available, but it remains unclear whether they are correlated with stroke risk in the community-dwelling population. We aimed to examine the longitudinal association of the triglyceridemic-waist phenotypes, the triglyceride glucose (TyG) index, as well as TyG-related indicators with stroke risk.

**Methods:**

In this study, we conducted a prospective cohort study in Hunan, a region located in Central China, where the prevalence of stroke is relatively high. We included a total of 20185 subjects aged ≥40 years between November 2017 and December 2018. Triglyceride glucose-body mass index (TyG-BMI) and triglyceride glucose-waist circumference (TyG-WC) were calculated as multiplying TyG index by BMI and WC, respectively. Triglyceride waist phenotypes were categorized into four phenotypes: HTGW (elevated triglyceride and enlarged WC), NTNW (normal triglyceride and normal WC); HTNW (high triglyceride and normal WC), and NTGW (normal triglyceride and enlarged WC). We constructed a multivariable Cox regression model to assess the association between these novel lipid indicators and the risk of stroke. Subgroup analysis was conducted to test the robustness of our research findings. ROC curve was used for assessing the predictive ability of different stroke risk indices.

**Results:**

After 2 years of follow- up, 135 participants experienced new stroke events. After adjusting for potential confounders, we found that participants with HTGW had higher likelihood of stroke (HR: 1.96, 95% CI: 1.21 to 3.16). However, we did not find significant associations for HTNW (HR: 1.42, 95% CI: 0.91 to 2.21) and NTGW (HR: 1.09, 95% CI 0.67 to 1.78). when compared to participants in the first TyG quartile, those in the fourth TyG quartile were associated with a 2.06-fold (95% CI: 1.22, 3.50) risk of stroke. Each 1-SD increase in TyG, TyG-BMI, and TyG-WC was associated with a higher risk of stroke, with adjusted HRs of 1.34 (95% CI: 1.11 to 1.61), 1.35 (95% CI: 1.14 to 1.59), and 1.23 (95% CI: 1.04 to 1.46), respectively. In subgroup analyses, those positive relationships appeared to be stronger among male participants with lower levels of physical activity and smoking.

**Conclusion:**

HTGW, along with higher levels of TyG and TyG-related indicators, were found to be associated with an elevated risk of stroke. HTGW and these novel lipid indicators might be reliable indicators to identify populations at elevated risk of stroke.

## Background

Globally, stroke is a major contributor to disability and the second leading cause of death (1). In China, the prevalence of stroke continues its upward trajectory. According to a comprehensive study involving a sizeable and representative sample of adults aged 40 years or older, the estimated incidence, prevalence, and mortality rate of stroke in China in 2020 were 505.2 per 100000 person-years, 2.6%, and 343.4 per 100000 person-years, respectively (2). It’s noteworthy that obesity is an established and modifiable risk factor for stroke (3). Obesity-related indices have become important tools for assessing the risk of cardiovascular disease (CVD) in population due to their cost-effectiveness and convenience (4). However, there is still controversy regarding the predictive power of different obesity indicators (5).

Adipose tissue, a pivotal biomarker for obesity, not only influences insulin resistance but also body weight, blood pressure, lipids, coagulation, and inflammation, thereby contributing to the development of stroke and other cardiovascular disease (6). Body mass index (BMI) and waist circumference (WC) have been used as proxies for obesity and CVD risk for many years (5, 6). However, WC and BMI alone have been criticized for their inability to differentiate body composition and metabolic profiles, making them ineffective for predicting cardiometabolic risk (7). In recent years, novel lipid indicators based on a combination of anthropometric measures and biochemical measures have been established and were considered superior to traditional anthropometric parameters (8). The combination of elevated triglycerides and WC, known as hypertriglyceridemic-waist phenotypes, provides a better evaluation of excess visceral adiposity (9) and predicts incident coronary heart disease (10). The triglyceride glucose (TyG) index and the combination of TyG with adiposity status (TyG-BMI and TyG-WC) were considered as simple and clinically useful surrogate markers for insulin resistance and metabolic health (11). These emerging indicators, characterized by their simplicity, enhanced precision, and pragmatic applicability, hold the potential to proficiently mitigate cardiometabolic risk among police personnel, offering a promising approach to reducing the incidence of cardiometabolic diseases.

While prior studies have indicated that novel lipid indicators are risk factors for developing CVD events, this evidence regarding their association with the risk of stroke has been limited and the findings have been inconsistent (12–15). A cross-sectional study including 2830 elderly adults from the Northern Shanghai Study identified no significant association between the TyG Index and macrovascular damage (16). In addition, the study by Alizargar J et al. showed that the TyG index, when used for diagnosing and predicting cardiovascular disease, might be biased and provide lower value than expected (17). To date, only one prospective study has revealed the relationship between ischemic stroke and hypertriglyceridemic-waist phenotypes (18). However, this study was conducted on a small-scale and rural population. A cross-sectional investigation involving a cohort of 2,296 adult participants from Turkey revealed that HTGW phenotype exhibited predictive capacity for coronary artery disease (CAD) risk in the male subgroup, while such predictability is not evident among female participants (19). Conversely, a cohort study involving 6834 participants showed that the HTGW phenotype was a crucial predictor of CVD only among women (20). The variations in results across different research designs and study populations have raised questions about their credibility. Hence, there is a need for further investigation into the relationship between hypertriglyceridemic-waist phenotypes and TyG as well as TyG-related parameters and the risk of stroke in the general population. Moreover, the number of prospective studies addressing this issue is limited for the Chinese population. Given the diverse effects of epidemiological and metabolic risk factors in different populations and the different lifestyles in different regions (21), therefore, this population-based study in Central China was performed to examine the association of novel lipid indictors and 2-years stroke risk. We also performed subgroup analyses to assess the robustness of the findings.

## Methods

### Study design and participants

The data used in our study were obtained as part of the China Stroke High-risk Population screening and Intervention Program (CSHPSIP), an ongoing population-based screening project launched by the Chinese government in mainland China. It aimed to reduce the risk of stroke and control rates of stroke risk factors through screening, physical examinations, and comprehensive interventions. Details regarding the design, survey methods, and objectives have been outlined and recommended (22). The enrollment criteria for community-dwelling adults were as follows: community residents aged ≥40 years (residence for 6 months) and provision of informed consent. The survey protocol was reviewed and approved by the Ethics Committee of the participating hospitals.

Between 2017 to 2018, 54338 participants received a face-to-face interview using a standardized protocol in the twenty-six communities in Hunan province (including thirteen urban areas and thirteen rural areas). Follow-up interventions for screened populations were performed every 2 years after the baseline assessment. The links between the baseline data set and the second intervention data set links were established through unique identification card number matching. All subjects with stroke (including ischemic stroke, intracerebral hemorrhage, and stroke of undetermined type) were recorded. The diagnosis of stroke required patients to provide an imaging certificate (MRI/CT) or a diagnosis certificate from a secondary or higher medical unit (2). The diagnosis of stroke was made according to the International Classification of Diseases, 10th version (ICD-10). Stroke were classified as ischemic stroke [IS, ICD 63], intracerebral hemorrhage [ICH, ICD 61], subarachnoid hemorrhage [SAH, ICD 60], and stroke of undetermined type. It was evaluated by independently by two trained neurologists and discrepancies in diagnosis were resolved through consensus. Any strokes that occurred during the survey period were considered incident cases. Individuals who did not undergo follow-up interventions within 2 years were excluded. In this study, we further excluded individuals with missing data on the anthropometric measures (n=76) or biochemical measures (n=226). A total of 20185 subjects were included in the final analysis (**Figure S1**).

### Baseline data collection and definitions

Trained interviewers administered a comprehensive questionnaire to collect information on demographic characteristics (age, sex), education level, lifestyle risk factors (alcohol drinking and smoking habits), medical history (hypertension, diabetes mellitus, stroke, and atrial fibrillation), family medical history (hypertension, diabetes, and coronary heart disease), and physical examinations. Physical activity was defined as engaging in regular physical exercise for at least 1 year, >2 times per week, and >30 minutes each time (23), or involving heavy physical labor according to WHO recommendations standards (24). Alcohol consumption was categorized into three groups: heavy alcohol consumption (defined as the intake of alcoholic beverages ≥3 times per week and ≥100 mL per drinking episode), light to moderate alcohol consumption (defined as the intake of alcoholic beverages <3 times per week or <100 mL per drinking episode), and none (2). Smoking was defined as continuous or cumulative smoking for >6 months. Education status was categorized as primary school or below, middle school, and high school or further education. Diabetes mellitus was defined by: (1) fasting plasma glucose (FPG) ≥7.0 mmol/L; (2) self-reported diabetes mellitus; (3) use of oral hypoglycemic agents or insulin injections. Hypertension was defined as: (1) systolic blood pressure (SBP) ≥140 mm Hg and/or diastolic blood pressure (DBP) ≥90 mm Hg; (2) self-reported hypertension; (3) use of antihypertension medications. Atrial fibrillation was defined as self-reported history of persistent atrial fibrillation or documented electrocardiogram (ECG) results. Because the primary outcome was new stroke events during the 2-year of follow-up period, in order to provide a comprehensive exploration of novel lipid indicators and their association with the risk of stroke, individuals with a history of stroke were not excluded in our analysis.

Body weight, height, waist circumference (WC), fasting blood glucose and blood pressure were measured and recorded by trained nurses or physicians following standard protocols. The removal of shoes and hats and wearing lightweight clothing were required when measuring the height and weight of the participants. Height and weight measurements were accurate to 0.1 cm and 0.1 kg, respectively. WC was measured with a tapeline to the nearest 0.1 cm at the midpoint between the lower margin costal arch and anterior superior iliac spine at the end of expiration (25). Blood pressure was measured twice consecutively with a 2-minute interval after resting for at least 15 min, and the average of the two measurements was taken as the final result. Fasting plasma and serum samples were collected to measure low-density lipoprotein cholesterol (LDL-C), high-density lipoprotein cholesterol (HDL-C), total cholesterol (TC), triglycerides (TG), and Hemoglobin A1c (HbA1c) using an HP-AFS/3 automatic immunoassay system A3 Specific Protein Analyzer with supporting reagents (Shijiazhuang Hebo Biotechnology Co., Ltd., Shijiazhuang, China). Body mass index (BMI) was calculated as weight in kilograms divided by the square of height in meters (26). According to the recommended criteria for hypertriglyceridemic-waist phenotypes (9, 27), normal TG levels were defined as less than 1.7 mmol/L, and normal WC was defined as less than 90 cm for men and less than 85 cm for women. Participants were categorized into four groups: (1) normal TG levels and normal WC (NTNW); (2) high TG levels and normal WC (HTNW); (3) normal TG levels and enlarged WC (NTGW); (4) high TG levels and enlarged WC (HTGW). TyG and TyG-related indicators were calculated using established formulas (**Table S1**).

### Statistical analysis

Categorical variables were summarized with counts and percentages; while continuous variables were presented as either mean± standard deviation (SD) or median (interquartile range), depending on the data distribution. Baseline characteristics for participants were presented based on the presence of incident stroke within 2 years and were compared using Student’s t-test or the Wilcoxon rank-sum test for continuous variable and the χ² test for categorical variables.

The novel lipid indicators TyG, TyG-BMI, and TyG-WC were transformed into categorical variables by using quartile methods. The association between stroke risk and triglyceridemic-waist phenotypes, TyG and TyG-related indicators was assessed using Cox regression models. For individuals with a prior history of stroke, their baseline entry time was defined as the start of their participation in the study, rather than the time of their previous stroke event. Subsequently, we tracked their observation period for 2 years from the baseline entry date to determine if a new stroke event occurred. Hazard ratios (HRs) with 95% confidence intervals (CIs) were calculated with or without adjustment for covariates. Model 1 was unadjusted. Model 2 was adjusted for age, sex, education, smoking, alcohol drinking, physical activity, family history (hypertension, diabetes, and coronary heart disease), and medical history (hypertension, stroke, atrial fibrillation, and diabetes). We also evaluated the risk of new stroke events associated with one SD increase in lipid-related indicators. The discriminative power of TyG-related parameters and TyG for stroke risk was evaluated using receiver operating characteristic (ROC) analysis. Subsequently, pairwise comparisons of the areas under the receiver operating characteristic curves (AUCs) were made between TyG, TyG-related indicators, and established biomarkers including BMI and WC. A P-value<0.05 (two-tailed) was considered statistically significant.

Stratified analysis was conducted by sex (male or female), age group (< 60 or ≥ 60 years), smoking (yes or no), physical activity (inactive or active), history of stroke (yes or no), history of hypertension (yes or no), and history of diabetes (yes or no). The multiplicative interaction of triglyceridemic-waist phenotypes and lipid-related indicators with sex, age, smoking, and medical history was estimated respectively using likelihood ratio test. To confirm the robustness of the primary analysis findings, we performed sensitivity analyses by excluding subjects with a history of stroke (n=734), and reran the models. All data management and statistical analyses were conducted using the statistical software SPSS version 25.0 (IBM SPSS, Armonk, NY, USA) and R version 4.2.1 (R Development Core Team, Vienna, Austria). P<0.05 was considered statistically significant.

## Results

### Baseline characteristic descriptions

In the present study, a total of 20185 subjects were included, of which 135 subjects were classified into the stroke group and 20050 into the non-stroke group (**Table 1**). The baseline characteristics of the included subjects did not significantly differ from those of the excluded subjects (**Table S2**). Over the 2-year follow-up period, 226 participants passed away. Compared to those without stroke, individuals who developed new stroke events tended to be older, smokers, and less physically active. Moreover, history of hypertension, diabetes, and stroke showed significant differences between cases and controls. They also had higher systolic blood pressure (SBP), diastolic blood pressure (DBP), fasting plasma glucose (FPG), triglycerides (TG), and glycosylated hemoglobin (HbA1c) than those without new stroke events (all P-values <0.05). More participants in the stroke group than in the non-stroke group were in the elevated triglyceride and enlarged waist circumference (HTGW) (18.5% vs 10.2%), normal triglyceride and enlarged waist circumference (NTGW) (17.0% vs 16.5%), and high triglyceride and normal waist circumference (HTNW) (22.2% vs 20.3%) group (P-values = 0.006). A higher level of the triglyceride glucose index (TyG), Triglyceride glucose-body mass index (TyG-BMI), and triglyceride glucose-waist circumference (TyG-WC) at baseline in stroke group was significant than in non-stroke group (P-values <0.05). There was no significant difference in gender, education, drinking habits, history of atrial fibrillation, and family history between the stroke and non-stroke group (**Table 1**).

**Table 1:** Baseline characteristics of participants according to the cerebrovascular disease status at follow-up.

Variables	Total (n = 20185)	New stroke events (n = 135)	Non-case (n = 20050)	P-value
Age, years	59.34 ± 10.99	62.90 ± 9.40	59.32 ± 11.00	<0.001*
Gender, n (%)				0.129
Male	8863 (43.9)	68 (50.4)	8795 (43.9)	
Female	11322 (56.1)	67 (49.6)	11255 (56.1)	
Education, n (%)				0.059
Primary school or below	7423 (36.8)	61 (45.2)	7362 (36.7)	
Middle school	7311 (36.2)	48 (35.6)	7263 (36.2)	
High school or further location	5451 (27.0)	26 (19.3)	5425 (27.1)	
Smoker, n (%)	4202 (20.8)	39 (28.9)	4163 (20.8)	0.020*
Drinking, n (%)				0.467
Never	16898 (83.7)	108 (80.0)	16790 (83.7)	
Light drinking	569 (2.8)	4 (3.0)	565 (2.8)	
Heavier drinking	2718 (13.5)	23 (17.0)	2659 (13.4)	
Family history, n (%)				
Hypertension	4441 (22.0)	37 (27.4)	4404 (22.0)	0.125
Diabetes	1554 (7.7)	11 (8.1)	1543 (7.7)	0.585
Coronary heart disease	1818 (9.0)	11 (8.1)	1807 (9.0)	0.930
Medical history, n (%)				
Hypertension	7298 (36.2)	86 (63.7)	7212 (36.0)	<0.001*
Diabetes mellitus	4313 (21.4)	46 (34.1)	4267 (21.1)	<0.001*
Atrial fibrillation	161 (0.8)	2 (1.5)	159 (0.8)	0.370
Stroke	734 (3.6)	24 (17.8)	710 (3.5)	<0.001*
Physical activity, n (%)				0.020*
Inactive	5395 (26.7)	48 (35.6)	5347 (26.7)	
Active	14790 (73.3)	87 (64.4)	14703 (73.3)	
SBP, mmHg	128.00 (119.00, 137.00)	133.00 (122.00, 150.00)	128.00 (119.00, 136.00)	<0.001*
DBP, mmHg	78.00 (72.00, 82.50)	80.00 (74.00, 85.00)	78.00 (72.00, 82.00)	0.026*
FPG, mmol/L	5.26 (4.70, 5.99)	5.59 (4.90, 6.86)	5.26 (4.70, 5.98)	0.003*
Triglyceride, mmol/L	1.41 (1.07, 1.86)	1.54 (1.12, 2.21)	1.41 (1.07, 1.85)	0.009*
Total cholesterol, mmol/L	4.62 ± 1.03	4.79 ± 0.96	4.61± 1.03	0.055
HDL-C, mmol/L	1.31 (1.10, 1.58)	1.24 (1.05, 1.61)	1.31 (1.10, 1.58)	0.324
LDL-C, mmol/L	2.62 ± 0.81	2.67 ± 0.85	2.61 ± 0.81	0.443
HbA1c (%)	5.40 (5.00, 5.90)	5.60 (5.20, 6.20)	5.40 (5.00, 5.90)	0.015*
Body mass index	23.50 ± 2.62	24.00 ± 2.81	23.50 ± 2.62	0.027
Waist circumference, cm	81.00 (76.00, 87.00)	83.00 (78.00, 90.00)	81.00 (76.00, 87.00)	0.017*
Triglyceridemic-waist phenotypes, N (%)				0.006*
NTNW	10694 (53.0)	10576 (53.1)	57 (42.2)	
NTGW	3320 (16.4)	3281 (16.5)	23 (17.0)	
HTNW	4111 (20.4)	4048 (20.3)	30 (22.2)	
HTGW	2060 (10.2)	2026 (10.2)	25 (18.5)	
Novel lipid indicators				
TyG	9.41 ± 0.51	9.59 ± 0.53	9.41 ± 0.51	<0.001*
TyG-BMI	221.46 ± 29.30	230.44 ± 32.22	221.40 ± 29.27	<0.001*
TyG-WC	765.98 (706.87, 830.12)	789.74 (729.74, 867.61)	765.83 (706.79, 829.83)	0.001*

Data are summarized as number (percentage), mean ± standard deviation, or median (interquartile range).

WC, waist circumference; BMI, body mass index; SBP, systolic blood pressure; DBP, diastolic blood pressure; FPG, fasting plasma glucose; TC, total cholesterol; TG, triglycerides; HDL-C, high-density lipoprotein cholesterol; HbA1c, glycosylated hemoglobin; TyG, triglyceride glucose; NTGW normal triglyceride level and enlarged waist circumference; NTNW normal triglyceride level and normal waist circumference. HTGW elevated triglyceride level and enlarged waist circumference; HTNW elevated triglyceride level and normal waist circumference. *P < 0.05.

### Independent association of HTGW and lipid indicators on the risk of stroke

**Figure 1:**
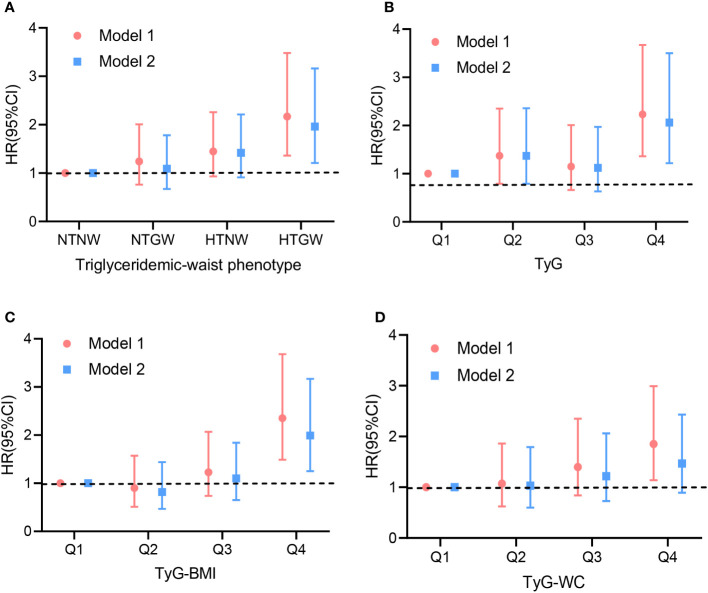
Hazard ratios for the association between lipid-related indices and stroke in population. Model 1: crude model. Model 2: adjusted for age, sex, education, smoking, alcohol drinking, physical activity, family history (hypertension, diabetes, and coronary heart disease) and medical history (hypertension, diabetes mellitus, stroke, and atrial fibrillation). **(A)** triglyceridemic-waist phenotypes; **(B)** triglyceride glucose (TyG); **(C)** Triglyceride glucose-body mass index (TyG-BMI); **(D)** Triglyceride glucose-waist circumference (TyG-WC). HR, hazard ratio; CI, confidence interval.

As was shown in **Table 2** and **Figure 1**, compared with the subjects in the NTNW group, participants in the HTGW group had an increased risk of stroke (Hazard ratios [HR]: 2.17, 95% confidence interval [CI]: 1.36-3.48). After adjusting for various potential confounding factors, including age, sex, education, smoking, alcohol drinking, physical activity, family history (hypertension, diabetes, and coronary heart disease), and medical history (hypertension, stroke, atrial fibrillation, and diabetes), this significant association remained (HR: 1.96, 95% CI: 1.21-3.16). The risk of stroke increased with the increment of TyG, TyG-BMI, and TyG-WC (**Table 2**). After adjusting for all covariates, each 1-unit increment in TyG, TyG-BMI, and TyG-WC was associated with 34% (HR: 1.34, 95% CI: 1.11-1.61), 35% (HR: 1.35, 95% CI: 1.14-1.59), and 23% (HR: 1.23, 95% CI: 1.04-1.46) increased risk of stroke, respectively. Compared to the lowest quartile of the indices, the highest quartile of TyG, TyG-BMI, and TyG-WC was associated with a 2.23-fold (95% CI: 1.36-3.67, P_-trend_ = 0.002), 2.35-fold (95% CI: 1.49-3.68, P_-trend_ = 0.001), and 1.85-fold (95% CI: 1.14-2.99, P_-trend_ = 0.005) risk of stroke. After adjusting for all covariates, the highest quartile of TyG remained associated with the risk of stroke (HR: 2.06, 95% CI: 1.22-3.50, P_-trend_ = 0.012). The results of the ROC analysis were presented in **Table S3** and **Figure S2**. The AUC for the TyG index, TyG-BMI index, and TyG-WC index in predicting stroke risk was 0.589, 0592, and 0.585, respectively. The Delong test revealed no statistically significant difference between the predictive abilities of these indexes for stroke risk (all p > 0.05). However, TyG-BMI demonstrated a higher predictive ability for stroke risk than BMI using the Delong tests (p=0.014). Similarly, TyG-WC performed better than WC in predicting stroke risk (p=0.037).

**Table 2:** Association between lipid indicators and the risk of stroke.

	N*		HR (95% CI)	
	Non-stroke group	Stroke group	Crude	Adjusted†
Triglyceridemic-waist phenotype				
NTNW	10637	57	1.00	1.00
NTGW	3297	23	1.24(0.76,2.01)	1.09(0.67,1.78)
HTNW	4081	30	1.45(0.93,2.26)	1.42(0.91,2.21)
HTGW	2035	25	2.17(1.36,3.48)	1.96 (1.21,3.16)
TyG				
Quartile 1	4858	22	1.00	1.00
Quartile 2	5095	28	1.37(0.79,2.35)	1.37(0.79,2.36)
Quartile 3	5120	32	1.15(0.66,2.01)	1.12(0.63,1.97)
Quartile 4	4977	53	2.23(1.36,3.67)	2.06(1.22,3.50)
P for trend			0.002	0.012
Every 1-unit increment			1.38(1.16,1.63)	1.34(1.11,1.61)
TyG-BMI				
Quartile 1	5025	21	1	1
Quartile 2	5018	29	0.90(0.51,1.57)	0.82(0.47,1.44)
Quartile 3	5016	29	1.23(0.74,2.07)	1.10(0.65,1.84)
Quartile 4	4991	56	2.35(1.49,3.68)	1.99(1.25,3.17)
P for trend			0.001	0.001
Every 1-unit increment			1.43(1.21,1.68)	1.35(1.14,1.59)
TyG-WC				
Quartile 1	5018	25	1	1
Quartile 2	5022	26	1.07(0.62,1.86)	1.03(0.60,1.79)
Quartile 3	5012	35	1.40(0.84,2.35)	1.22(0.73,2.06)
Quartile 4	4997	49	1.85(1.14,2.99)	1.47(0.89,2.43)
P for trend			0.005	0.087
Every 1-unit increment			1.34(1.14,1.58)	1.23(1.04,1.46)

WC, waist circumference; BMI, body mass index; TyG, triglyceride glucose; NTGW, normal triglyceride level and enlarged waist circumference; NTNW, normal triglyceride level and normal waist circumference. HTGW elevated triglyceride level and enlarged waist circumference; HTNW elevated triglyceride level and normal waist circumference; HR, hazard ratio; CI, confidence interval.

* N represents sample size for non- stroke group or for stroke group.

† Adjustment for age, sex, education, smoking, alcohol drinking, physical activity, family history (hypertension, diabetes, and coronary heart disease) and medical history (hypertension, diabetes mellitus, stroke, and atrial fibrillation).

### Stratified analysis and sensitivity analysis

The multiple-adjusted HRs for new stroke events among participants associated with triglyceridemic-waist phenotypes varied according to age, sex, physical activity, smoking, history of stroke, history of diabetes, and history of hypertension (**Table S4**, **Figure S3**). Compared to the NTNW group, participants with HTGW were significantly associated with an increased risk of stroke in stratified groups including males, those with inactive physical activity, smokers, and those without a history of hypertension or stroke or with a history of diabetes. No significant interaction was observed between HTGW and age, sex, physical activity, smoking, history of stroke, history of diabetes, or history of hypertension (**Table S4**).

In subgroup analysis, the positive relationship of all the novel lipid indicators with the risk of stroke remained significant in subjects with inactive exercise and smoking (**Figure S4**). Compared to subjects with a history of diabetes and stroke, a stronger association of TyG with stroke was found in the subgroup without a history of diabetes and stroke. Significant interactions were observed between TyG-BMI and TyG-WC and age, smoking, and history of hypertension regarding the risk of stroke (**Figure S4**, all P value for interaction < 0.05). Sensitivity analysis was performed after excluding subjects with a history of stroke. Similar results on the association between novel lipid indicators and stroke risk were found, demonstrating the robustness of the findings in the primary analysis (**Table S5**).

## Discussion

**Figure 2:**
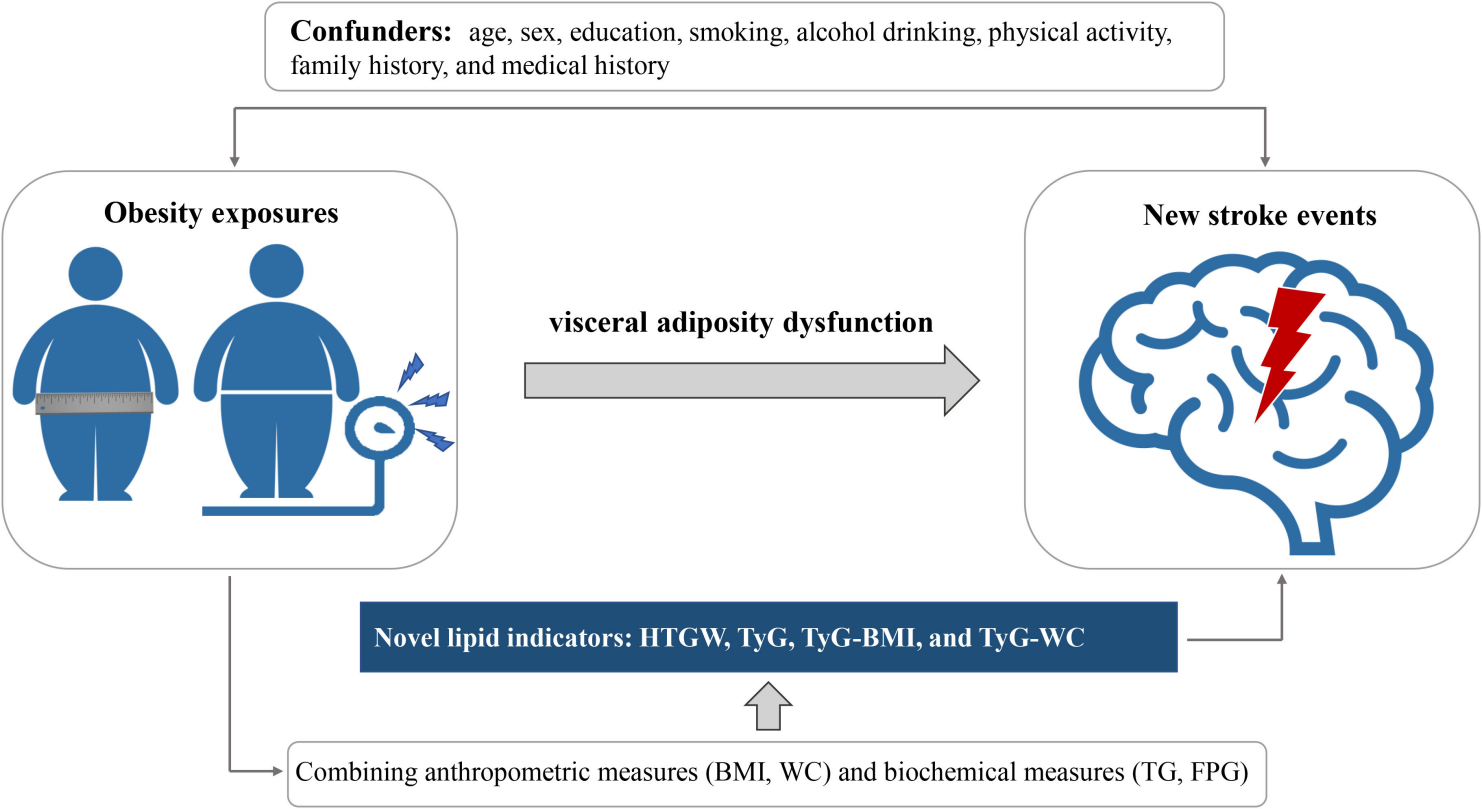
Graphical abstract for this study. WC, waist circumference; BMI, body mass index; FPG, fasting plasma glucose; TG, triglycerides; TyG, triglyceride glucose; HTGW elevated triglyceride level and enlarged waist circumference.

Given the heavier burdens of metabolic risk factors in Central China, our findings, based on a large population-based prospective cohort study, indicate that HTGW is associated with an increased risk of stroke, while TyG, TyG-BMI, and TyG-WC are positively associated with the risk of stroke (**Figure 2**). Further subgroup analysis reveals that HTGW and the novel lipid indicators remain stable risk factors for stroke in male subjects with inactive exercise and smoking.

Currently, only magnetic resonance imaging (MRI) and computed tomography (CT) can accurately assess the content of visceral fat (28). However, due to concerns about radiation exposure and high costs, these imaging techniques are not practical for large-scale population screening. HTGW, defined as the coexistence of an elevated WC and hypertriglyceridemia, serves as a straightforward marker of visceral adiposity (9). In recent years, previous studies have consistently indicated that HTGW was an independent risk factor for conditions such as hypertension, diabetes, and cardiovascular diseases (18, 27, 29–31). A prospective study conducted within the Chinese rural population highlighted that HTGW was significantly associated with an elevated risk of ischemic stroke and could be a practical tool for screening individuals with an increased risk of ischemic stroke (18). Moreover, data from a study involving 95015 participants (ranging from 18 to 98 years old) in the Kailuan Study showed that the HTGW phenotype was independently associated with the risk of CVDs (29). Consistently, in our study, we also found that HTGW was associated with a higher risk of stroke, which further supported previous research finding that the risk of cardiovascular disease depended jointly on participants’ body size and metabolic profile (32). Additionally, our stratified analysis revealed that the HTGW phenotype remained a stable risk factor for stroke in subjects with inactive physical activity and smoking.

TyG, a novel indicator determined by FPG and TG, has gained recognition as a valuable predictor of T2DM, metabolic disorders, cardiovascular and atherosclerotic disease (33–35). Evidence suggests that the occurrence and development of stroke is accompanied by an increase in a range of metabolic indices, including BMI, WC, and lipid profiles (36). However, the evidence across published studies on the relationship between WC, BMI and the CVD risk presents inconsistencies (37). Data sourced from the Clinical Practice Research Datalink and Hospital Episode Statistics databases from United Kingdom showed that higher BMI was associated with a more favorable prognosis for subsequent cardiovascular events and mortality (38). Similarly, a study involving 67086 patients with T2DM also found an inverse association between BMI and the risk of stroke (39). Conversely, several studies indicated that BMI and WC were positively associated with the CVD risk (40–42). Different explanations were proposed to explain these paradoxical findings. It was suggested that BMI was not a perfect indicator of obesity, as it neither captures the distribution of body fat nor distinguished between total fat mass and lean mass (43). Additionally, serum lipid profiles were involved in the pathophysiological mechanisms of CVD events (44). Therefore, our study derived a series of complex indicators including TyG, TyG-BMI, TyG-WC that can reflect cardiometabolic level. It has been indicated that lipid-related indices such as TyG, TyG-BMI, TyG-WC (involving BMI, WC, TG, and FPG) might provide a more comprehensive evaluation of metabolic disorders and more efficient compared to traditional measures like BMI and WC (5, 45, 46). In our study, we found that TyG and TyG-related indicators were positively associated with the risk of stroke and may be an easily measured and reliable tools for identifying individuals at high risk of stroke.

Previously, the results from stratified analyses examining the association between the TyG index and the risk of CVD were inconsistent. A study based on 5014 patients from the Vascular Metabolic CUN cohort (VMCUN cohort) revealed that TyG was a risk factor for CVD events in individuals without diabetes, yet it lost its predictive value in subjects with diabetes (47). While another cohort study focused on elderly acute coronary syndrome patients demonstrated that the predictive value of the TyG index was observed only among subjects with diabetes (48). Such inconsistencies might be attributed to the influence of hypoglycemic drugs blood glucose levels and therewith affecting the TyG index. In addition, traditional cardiovascular risk factors, including elevated LDL cholesterol levels, high blood pressure, and smoking, exert a more significant impact on CVD events compared to lipid-related factors (49). Through our stratified analysis, we have found that the TyG index hold promise as a screening tool for stroke risk, particularly in subjects without history of diabetes. This finding extends the conclusions drawn from previous studies conducted in non-diabetic populations (35) and health adults (47). Moreover, studies on TyG and CVD risk were mostly focused on elderly individuals and the predictive value in young subjects was uncertain (50). In our subgroups, we found that the positive association between TyG and TyG-BMI and stroke risk appears to be more pronounced in elderly subjects. Given the uncertain predictive value of the TyG index in population, further studies based on larger prospective cohorts should be conducted.

Visceral adiposity dysfunction, which contributes to insulin resistance (IR) is an independent risk factor for CVD and can promote metabolic diseases such as hypertension, hyperlipidemia, obesity, and diabetes, and these metabolic disorders are frequently observed in high-risk groups for CVD (51). The lipid indicators including HTGW, TyG, TyG-WC, and TyG-BMI, which combine blood glucose, TG levels, BMI, and WC, are regarded as reliable indicators for assessing visceral adiposity dysfunction and IR (52). Although the exact mechanisms underlying the relationship between these indicators and stroke are unclear, several hypotheses have been proposed. Firstly, an imbalance between the metabolism of glucose and lipids, leading to oxidative stress and inflammation, causes atherosclerosis to appear and develop, and accelerates the evolution of cardiovascular disease (53). Secondly, visceral adiposity dysfunction can induce increased production of glycosylated products and free radicals, which contribute to an increase in reactive oxygen species (ROS) production, lead to collagen deposition and smooth muscle cell proliferation, and cause vascular endothelial injury (54). Third, IR can reduce the platelets’ responsiveness to the anti-aggregating effects of prostaglandins I2 and nitric oxide (NO) (55). This alteration can result in an excessive activation of platelets, fostering the onset of thrombosis and inflammation. These processes collectively contribute to the occurrence of unfavorable cardiovascular events (55). Moreover, there is a connection between insulin resistance and heightened engagement of the sympathetic nervous system, along with compromised cardiac autonomic function (56). These factors have been linked to the development of atherosclerotic cardiovascular disease.

This study represents the first investigation into the association between novel lipid indicators and stroke within the community population in Central China. Previous studies have found that obesity-related and lipid-related indicators were closely linked to CVD incidence in North and Eastern China (35, 57). However, there have been limited studies investigating the relationship between stroke incidence and lipid-related indicators in Central China. The geographical distribution of cardiovascular disease risk in China is intricate, with population risk levels exhibiting significant variations across regions (2). In Central China, the standardized rates of individuals at high risk of cardiovascular disease were notably elevated (10.7% [10.5-10.9]) (58). Among the seven major geographic regions of China (Northeast, North China, East China, South China, Southwest, Northwest, and Central China), the highest age-standardized prevalence and the second highest incidence and mortality of stroke was observed in Central China (59). Specifically, the incidence of stroke in Hunan Province is close to the incidence in central China (Hunan/Central China 333.6/326.1), which indicates that stroke in Hunan Province is a very serious burden, yet the risk is still increasing (60). In the factor analysis, the primary component corresponded to the obesity factor, with factor pattern loadings exceeding 0.9 for WC and BMI. The metabolic and physical activity factor also exhibited substantial loadings on blood lipid levels (0.7) and physical activity (0.5) (58). Given the substantial burden of metabolic risk factors and the heightened mortality and morbidity rates of CVD within the Central China population, we embarked on this study. Based on our study, we discovered that a clear positive relationship between lipid-related indicators and the risk of stroke, which is particularly evident in subjects with inactive exercise habits. These findings provide novel insights into the connection between obesity and the CVD risk among individuals with lack of exercise.

Being the most populous developing nation, China harbors a fifth of the global population and leads the world in stroke patient numbers. In response to the challenge of stroke, the China Stroke Prevention Project Committee (CSPPC) launched the China Stroke High-risk Population Screening and Intervention Program (CSHPSIP) as a critical national project in 2011 (22). It aimed to reduce the risk of stroke by enhancing the awareness and control rates of stroke risk factors among Chinese residents, promoting a healthy lifestyle, conducting public education campaigns, screening and physical examinations, and risk classification judgment, and improving medication adherence (61). These interventions in reducing stroke risk could potentially impact the association between baseline indicators and new stroke events, introducing the possibility of a masking effect that cannot be ruled out. To minimize the potential impact of these aforementioned effects on the current findings, we have conducted subgroup analyses to assess whether the relationship between baseline indicators and new stroke events varies among different subpopulations, taking into account potential interactions and moderation effects. In the future, it is important to extend the follow-up period beyond two years to assess the sustainability of the observed associations and determine whether the interventions have any lasting impact.

Our study has several limitations. First, the follow-up period was relatively short, and the number of outcomes events was small, which may invoke reverse causality and impact the establishment of a robust relationship between lipid-related indicators and stroke risk. Although, the relatively quick-onset state and the adjustment for a series of confounders minimizes the role of reverse causation, our follow-up period was still relatively short. Secondly, it’s important to note that all participants in our study were of Chinese ethnicity, so the generalizability of our findings to populations with different habits might be limited. In addition, the rate of participation in second round of screening in the current study was relatively low, which could potentially impact the reliability of our results to a certain extent. However, it’s worth noting that the baseline characteristics of subjects in the included and excluded groups did not display significant differences. Lastly, although our adjusted model aimed to control for confounding factors, there might still be other unknown variables that could influence the robustness of our findings. Therefore, the evidence is insufficient for making causal inferences based on the current study.

## Conclusions

The combined utilization of anthropometric measures (BMI and WC) along with biochemical measures (TG and FPG) to characterize obesity might provide improved discrimination of stroke risk compared to using each metric individually. HTGW and higher level of TyG, TyG-BMI, and TyG-WC were all found to be associated with an elevated risk of stroke. These novel lipid indicators, easily accessible in both epidemiological studies and clinical practice, have the potential to serve as effective and straightforward tools for assessing stroke risk.

## Data availability statement

The raw data supporting the conclusions of this article will be made available by the authors, without undue reservation.

## Ethics statement

The studies involving humans were approved by Capital Medical University Xuanwu Hospital (No. 2012045). The studies were conducted in accordance with the local legislation and institutional requirements. The participants provided their written informed consent to participate in this study.

## Author contributions

QH (1st Author): data curation, formal analysis, software, writing – original draft. YL (2nd Author): investigation, methodology, project administration, resources, validation, writing – review & editing. ZL (3rd Author): data curation, investigation, validation, writing – original draft. WM: methodology, software, writing – review & editing. JF: formal analysis, project administration, visualization, writing – review & editing. QH (6th Author): formal analysis, project administration, validation, writing – original draft. YL (7th Author): conceptualization, data curation, formal analysis, writing – original draft. JX: funding acquisition, resources, supervision, visualization, writing – review & editing. ZL (8th Author): writing - review & editing and validation.

## Supplementary material

Supplementary Figure 1 Flow chart of selection of participants in this study.

Supplementary Figure 2 The receiver operating characteristic curves of novel lipid indicators as markers to predict stroke risk. WC, waist circumference; BMI, body mass index; TyG, triglyceride glucose.

Supplementary Figure 3 Stratified analyses on the association between triglyceridemic-waist phenotypes and stroke risk. Adjustment for age, sex, education, smoking, alcohol drinking, physical activity, family history (hypertension, diabetes, and coronary heart disease) and medical history (hypertension, diabetes mellitus, stroke, and atrial fibrillation) except the corresponding stratification variable.

Supplementary Figure 4 Stratified analyses on the association between TyG, TyG-BMI, and TyG-WC and stroke risk. WC, waist circumference; BMI, body mass index; TyG, triglyceride glucose. Adjustment for age, sex, education, smoking, alcohol drinking, physical activity, family history (hypertension, diabetes, and coronary heart disease) and medical history (hypertension, diabetes mellitus, stroke, and atrial fibrillation) except the corresponding stratification variable.
