# Supplementary material for: Association of novel lipid indicators with the risk of stroke among participants in Central China: a population-based prospective study
Source: Front Endocrinol (Lausanne). 2023 Oct 2;14:1266552. doi: 10.3389/fendo.2023.1266552 (PMC10577285; doi:10.3389/fendo.2023.1266552)
Supplement: Supplementary file 1 [file Table_1.doc]

| **Supplementary Table 1. Published formula of novel lipid indicators** |
| --- |

| **IR Indexes** | **Sex** | **Formula** |
| --- | --- | --- |
| **TyG** | **Men/Women** | Ln [TG (mg/dL) x FPG (mg/dL)] |
| **TyG-BMI** | **Men/Women** | TyG x BMI |
| **TyG-WC** | **Men/Women** | TyG x WC |

WC, waist circumference; BMI, body mass index; TyG, triglyceride glucose index; TyG-BMI, triglyceride glucose-body mass indexp; TyG-WC, triglyceride glucose- waist circumference.
