# Supplementary material for: Association of novel lipid indicators with the risk of stroke among participants in Central China: a population-based prospective study
Source: Front Endocrinol (Lausanne). 2023 Oct 2;14:1266552. doi: 10.3389/fendo.2023.1266552 (PMC10577285; doi:10.3389/fendo.2023.1266552)
Supplement: Supplementary file 2 [file Table_2.docx]

**Supplementary Table 2. The baseline characteristics of the included and excluded participants.**

| **Variables** | **Total (n = 54338)** | **Included (n = 20185)** | **Excluded (n = 34153)** | **P-value** |
| --- | --- | --- | --- | --- |
| Age, years | 59.39±10.42 | 59.34±10.99 | 59.42±10.01 | 0.428 |
| Gender, n (%) |  |  |  | 0.751 |
| Male | 23907(44.0) | 8863(43.9) | 15044(44.0) |  |
| Female | 30431(56.0) | 11322(56.1) | 19109(56.0) |  |
| Education, n (%) |  |  |  | <0.001 |
| Primary school or below | 18859(34.7) | 7423(36.8) | 11436(33.5) |  |
| Middle school | 20857(38.4) | 7311(36.2) | 13546(39.7) |  |
| High school or further location | 14622(26.9) | 5451(27.0) | 9171(26.9) |  |
| Smoker, n (%) | 11002(20.2) | 4202(20.8) | 6800(19.9) | 0.111 |
| Drinking, n (%) |  |  |  | 0.013 |
| Never | 45805(84.3) | 16898(83.7) | 28907(84.6) |  |
| Light drinking | 1510(2.8) | 569(2.8) | 941(2.8) |  |
| Heavier drinking | 7023(12.9) | 2718(13.5) | 4305(12.6) |  |
| Family history, n (%) |  |  |  |  |
| Hypertension | 11251(20.7) | 4441(22.0) | 6810(19.9) | <0.001 |
| Diabetes | 3956(7.3) | 1554(7.7) | 2402(7.0) | 0.013 |
| Coronary heart disease | 4784(8.8) | 1818(9.0) | 2966(8.7) | 0.200 |
| Medical history, n (%) |  |  |  |  |
| Hypertension | 19469(35.8) | 7298(36.2) | 12171(35.6) | 0.223 |
| Diabetes mellitus | 11370(20.9) | 4313(21.4) | 7057(20.7) | 0.051 |
| Atrial fibrillation | 431(0.8) | 161(0.8) | 270(0.8) | 0.929 |
| Stroke | 1675(3.1) | 734(3.6) | 941(2.8) | <0.001 |
| Physical activity, n (%) |  |  |  | 0.284 |
| Inactive | 14380(26.5) | 5395(26.7) | 8985(26.3) |  |
| Active | 39958(73.5) | 14790(73.3) | 25168(73.7) |  |
| SBP, mmHg | 128.41±15.84 | 128.33±16.29 | 128.44±15.57 | 0.423 |
| DBP, mmHg | 77.80±8.25 | 77.72±8.78 | 77.84±7.92 | 0.140 |
| FPG, mmol/L | 5.69±1.60 | 5.69±1.74 | 5.68±1.52 | 0.616 |
| Triglyceride, mmol/L | 1.55±0.64 | 1.54±0.66 | 1.55±0.62 | 0.618 |
| Total cholesterol, mmol/L | 4.62±0.97 | 4.62±1.03 | 4.63±0.94 | 0.891 |
| HDL-C, mmol/L | 1.38±0.38 | 1.38±0.40 | 1.38±0.36 | 0.142 |
| LDL-C, mmol/L | 2.62±0.76 | 2.62±0.81 | 2.62±0.73 | 0.655 |
| Homocysteine, mmol/L | 11.55±3.62 | 11.53±4.37 | 11.55±3.09 | 0.571 |
| HbA1c (%) | 5.65±1.12 | 5.65±1.14 | 5.65±1.11 | 0.908 |
| Body mass index | 23.54±2.65 | 23.50±2.63 | 23.56±2.67 | 0.027 |
| Waist circumference, cm | 81.91±8.05 | 81.93±8.70 | 81.89±7.64 | 0.512 |
| Triglyceridemic-waist phenotypes, N (%) |  |  |  | 0.104 |
| NTNW | 28976(53.3) | 10694(53.0) | 18282(53.5) |  |
| NTGW | 8969(16.5) | 3320(16.4) | 5649(16.5) |  |
| HTNW | 10772(19.8) | 4111(20.4) | 6661(19.5) |  |
| HTGW | 5621(10.3) | 2060(10.2) | 3561(10.4) |  |
| Novel lipid indicators |  |  |  |  |
| TyG | 9.42±0.49 | 9.41±0.51 | 9.42±0.48 | 0.117 |
| TyG-BMI | 221.52±28.32 | 221.46±29.30 | 221.55±27.72 | 0.726 |
| TyG-WC | 771.02±95.08 | 771.25±96.76 | 770.88±94.07 | 0.665 |

Data are summarized as number (percentage) and mean ± standard deviation.

WC, waist circumference; BMI, body mass index; SBP, systolic blood pressure; DBP, diastolic blood pressure; FPG, fasting plasma glucose; TC, total cholesterol; TG, triglycerides; HDL-C, high-density lipoprotein cholesterol; HbA1c, glycosylated hemoglobin; TyG, triglyceride glucose; NTGW normal triglyceride level and enlarged waist circumference; NTNW normal triglyceride level and normal waist circumference. HTGW elevated triglyceride level and enlarged waist circumference; HTNW elevated triglyceride level and normal waist circumference.
