# Supplementary material for: Association of novel lipid indicators with the risk of stroke among participants in Central China: a population-based prospective study
Source: Front Endocrinol (Lausanne). 2023 Oct 2;14:1266552. doi: 10.3389/fendo.2023.1266552 (PMC10577285; doi:10.3389/fendo.2023.1266552)
Supplement: Supplementary file 3 [file Table_3.docx]

**Table S3. Area under curve the receiver operating characteristic curve for predicting** **stroke risk with novel lipid indicators.**

| Variables | AUC (95% CI) | ^a^ P value | ^b^ P value |
| --- | --- | --- | --- |
| WC | 0.560(0.430,0.686) | Reference | - |
| BMI | 0.567(0.474,0.666) | - | Reference |
| TyG | 0.589(0.467,0.700) | 0.377 | 0.488 |
| TyG-BMI | 0.592(0.496,0.703) | 0.227 | 0.014* |
| TyG-WC | 0.585(0.481,0.667) | 0.037* | 0.455 |

^a^P value: comparing the AUC of WC with TyG and TyG-related indexes.

^b^P value: comparing the AUC of BMI with TyG and TyG-related indexes.
