# Supplementary material for: Association of novel lipid indicators with the risk of stroke among participants in Central China: a population-based prospective study
Source: Front Endocrinol (Lausanne). 2023 Oct 2;14:1266552. doi: 10.3389/fendo.2023.1266552 (PMC10577285; doi:10.3389/fendo.2023.1266552)
Supplement: Supplementary file 4 [file Table_4.docx]

**Table** **S4. Stratified analyses on the association between triglyceridemic-waist phenotypes and the risk of stroke.**

|  | N^*^ | |  | HR (95% CI) ^†^ | | | | *P*-_interaction_ |
| --- | --- | --- | --- | --- | --- | --- | --- | --- |
|  | Non-stroke | stroke |  | NTNW | NTGW | HTNW | HTGW |  |
| Age |  |  |  |  |  |  |  | 0.393 |
| < 60 years | 10680 | 47 |  | 1.00 | 1.18(0.50,2.78) | 2.02(1.01,4.07) | 1.63(0.66,3.99) |  |
| ≥ 60 years | 9370 | 88 |  | 1.00 | 1.02(0.56,1.87) | 1.04(0.58,1.89) | 2.07(1.17,3.66) |  |
| Sex |  |  |  |  |  |  |  | 0.329 |
| Male | 8795 | 68 |  | 1.00 | 0.68(0.30,1.55) | 1.30(0.69,2.43) | 2.94(1.55,5.55) |  |
| Female | 11255 | 67 |  | 1.00 | 1.49(0.79,2.81) | 1.61(0.85,3.04) | 1.37(0.66,2.81) |  |
| Physical activity |  |  |  |  |  |  |  | 0.778 |
| Active | 14703 | 87 |  | 1.00 | 0.90(0.46,1.73) | 1.69(0.98, 2.94) | 1.48(0.78,2.82) |  |
| Inactive | 5347 | 48 |  | 1.00 | 1.52(0.72,3.23) | 0.72(0.27,1.93) | 2.73(1.32,5.67) |  |
| Diabetes |  |  |  |  |  |  |  | 0.975 |
| No | 15783 | 89 |  | 1.00 | 1.16(0.64,2.12) | 1.57(0.93,2.66) | 1.74(0.92,3.30) |  |
| Yes | 4267 | 46 |  | 1.00 | 0.90(0.38,2.06) | 1.07(0.46,2.48) | 2.17(1.03,4.54) |  |
| Stroke |  |  |  |  |  |  |  | 0.258 |
| No | 19340 | 111 |  | 1.00 | 1.45(0.85,2.43) | 1.68(1.04,2.73) | 2.06(1.19,3.57) |  |
| Yes | 710 | 24 |  | 1.00 | 0.16(0.02,1.25) | 0.47(0.11,1.97) | 1.46(0.54,3.95) |  |
| Smoking |  |  |  |  |  |  |  | 0.08 |
| No | 15887 | 96 |  | 1.00 | 1.15(0.66,2.02) | 1.43(0.86,2.39) | 1.32(0.71,2.44) |  |
| Yes | 4163 | 39 |  | 1.00 | 0.82(0.29,2.28) | 1.34(0.55,3.27) | 3.83(1.74,8.46) |  |
| Hypertension |  |  |  |  |  |  |  | 0.860 |
| No | 12838 | 49 |  | 1.00 | 1.16(0.50,2.72) | 1.09(0.51,2.36) | 2.47(1.14,5.39) |  |
| Yes | 7212 | 86 |  | 1.00 | 1.08(0.59,1.97) | 1.62(0.93,2.80) | 1.76(0.96,3.23) |  |

BMI, body mass index; HTGW, elevated triglyceride level and enlarged waist circumference; HTNW, elevated triglyceride level and normal waist circumference; NTGW, normal triglyceride level and enlarged waist circumference; NTNW, normal triglyceride level and normal waist circumference; HR, hazard ratio; CI, confidence interval.

^*^ N represents sample size for non- stroke group or for stroke group.

† Adjustment for age, sex, education, smoking, alcohol drinking, physical activity, family history (hypertension, diabetes, and coronary heart disease), and medical history except the corresponding stratification variable.
