# Supplementary material for: Association of novel lipid indicators with the risk of stroke among participants in Central China: a population-based prospective study
Source: Front Endocrinol (Lausanne). 2023 Oct 2;14:1266552. doi: 10.3389/fendo.2023.1266552 (PMC10577285; doi:10.3389/fendo.2023.1266552)
Supplement: Supplementary file 5 [file Table_5.docx]

**Table S5. Sensitivity analysis: adjusted hazard ratios and 95% confidence intervals for future stroke risk associated with baseline lipid indicators.**

|  | N* | |  | HR (95% CI) | |
| --- | --- | --- | --- | --- | --- |
|  | Non-stroke group | Stroke group |  | Crude | Adjusted† |
| Triglyceridemic-waist phenotype | | | | | |
| NTNW | 10314 | 43 |  | Ref. | Ref. |
| NTGW | 3143 | 22 |  | 1.59(0.95,2.66) | 1.44(0.85,2.43) |
| HTNW | 3948 | 27 |  | 1.72(1.06,2.78) | 1.68(1.04,2.72) |
| HTGW | 1935 | 19 |  | 2.21(1.29,3.80) | 2.06(1.19,3.57) |
| TyG |  |  |  |  |  |
| Quartile 1 | 4728 | 19 |  | Ref. | Ref. |
| Quartile 2 | 4950 | 24 |  | 1.20(0.66,2.19) | 1.26(0.69,2.30) |
| Quartile 3 | 4893 | 24 |  | 1.15(0.63,2.10) | 1.15(0.63,2.12) |
| Quartile 4 | 4769 | 44 |  | 2.16(1.26,3.70) | 2.07(1.17,3.68) |
| P for trend |  |  |  | 0.004 | 0.016 |
| Every 1-unit increment |  |  |  | 1.38(1.14,1.66) | 1.35(1.10,1.66) |
| TyG-BMI |  |  |  |  |  |
| Quartile 1 | 4896 | 26 |  | Ref. | Ref. |
| Quartile 2 | 4846 | 18 |  | 0.87(0.49,1.59) | 0.84(0.46,1.54) |
| Quartile 3 | 4821 | 22 |  | 1.06(0.60,1.88) | 0.98(0.55,1.74) |
| Quartile 4 | 4777 | 45 |  | 2.12(1.31,3.44) | 1.85(1.12,3.06) |
| P for trend |  |  |  | 0.001 | 0.009 |
| Every 1-unit increment |  |  |  | 1.38(1.15,1.64) | 1.31(1.09,1.58) |
| TyG-WC |  |  |  |  |  |
| Quartile 1 | 4903 | 20 |  | Ref. | Ref. |
| Quartile 2 | 4868 | 22 |  | 1.14(0.62,2.09) | 1.12(0.61,2.06) |
| Quartile 3 | 4805 | 30 |  | 1.53(0.87,2.70) | 1.39(0.79,2.47) |
| Quartile 4 | 4763 | 39 |  | 1.89(1.09,3.12) | 1.58(0.91,2.76) |
| P for trend |  |  |  | 0.011 | 0.075 |
| Every 1-unit increment |  |  |  | 1.34(1.13,1.61) | 1.26(1.05,1.51) |

WC, waist circumference; BMI, body mass index; TyG, triglyceride glucose; NTGW normal triglyceride level and enlarged waist circumference; NTNW normal triglyceride level and normal waist circumference. HTGW elevated triglyceride level and enlarged waist circumference; HTNW elevated triglyceride level and normal waist circumference; HR, hazard ratio; CI, confidence interval.

* N represents sample size for non- stroke group or for stroke group.

† Adjustment for age, sex, education, smoking, alcohol drinking, physical activity, family history (hypertension, diabetes, and coronary heart disease) and medical history (hypertension, diabetes mellitus, and atrial fibrillation).
