# Supplementary figures and images for: Association of novel lipid indicators with the risk of stroke among participants in Central China: a population-based prospective study
Source: Front Endocrinol (Lausanne). 2023 Oct 2;14:1266552. doi: 10.3389/fendo.2023.1266552 (PMC10577285; doi:10.3389/fendo.2023.1266552)

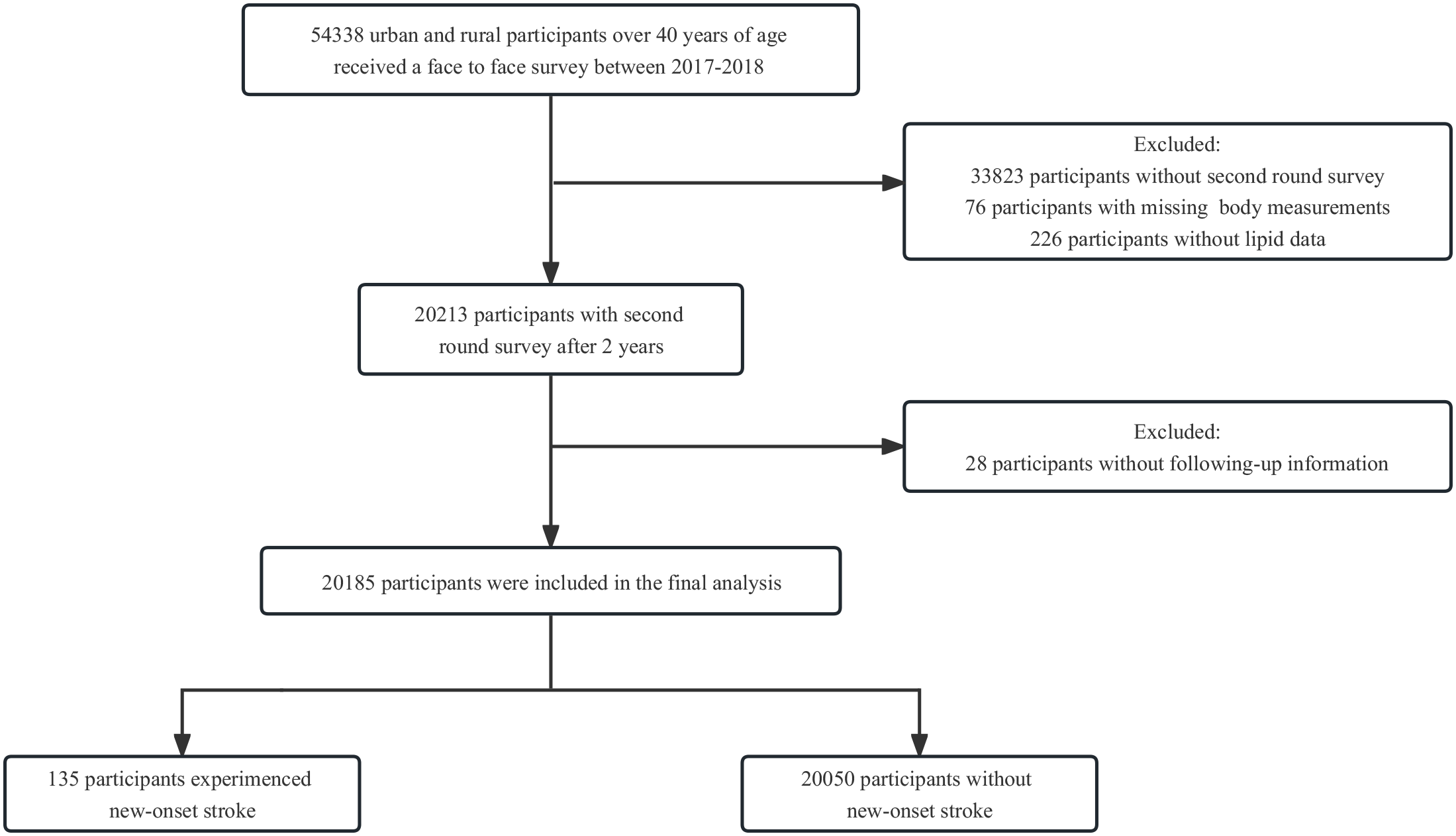

Supplement: Supplementary Figure 1 — Flow chart of selection of participants in this study. [file Image_1.tif]

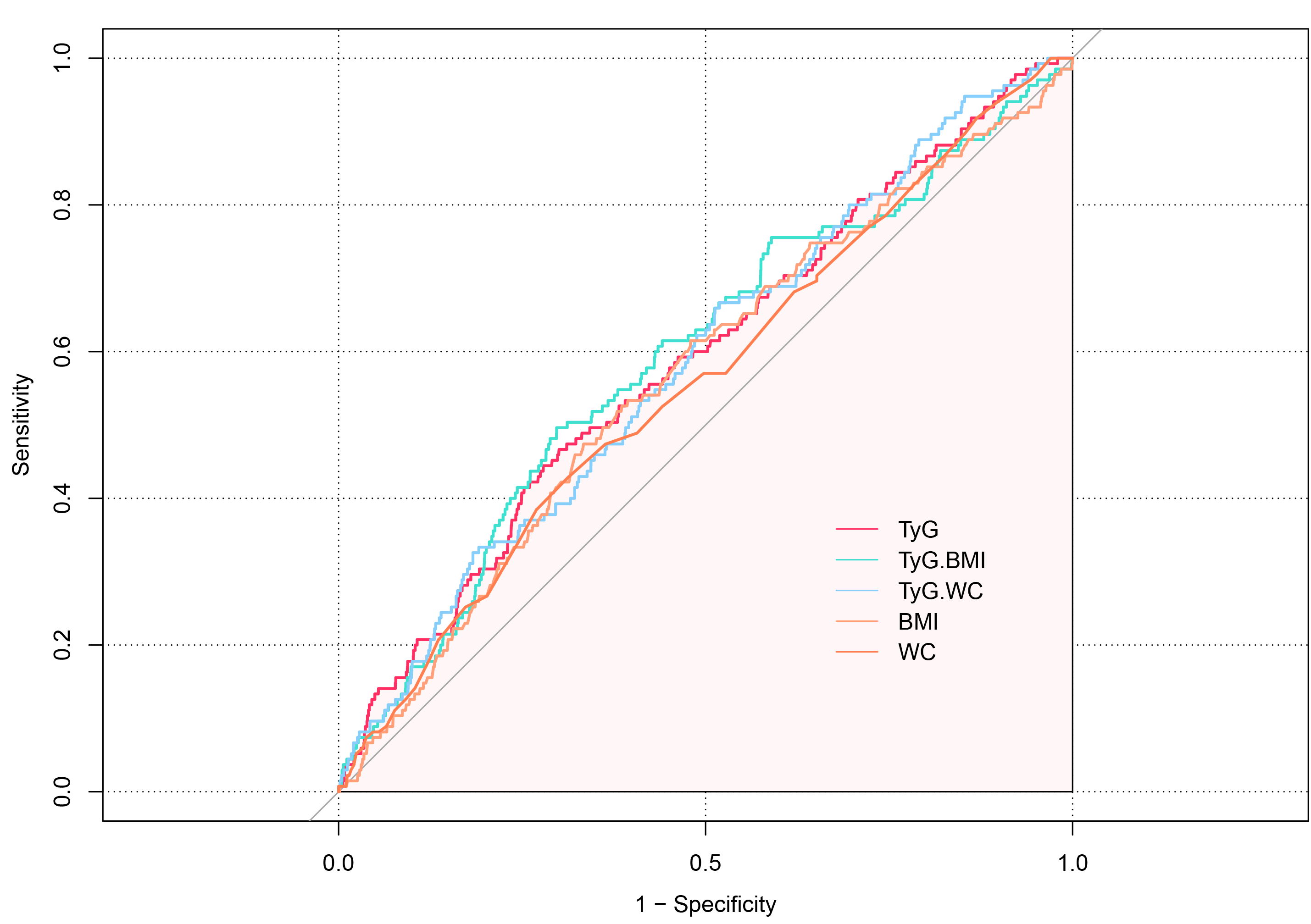

Supplement: Supplementary Figure 2 — The receiver operating characteristic curves of novel lipid indicators as markers to predict stroke risk. WC, waist circumference; BMI, body mass index; TyG, triglyceride glucose. [file Image_2.tif]

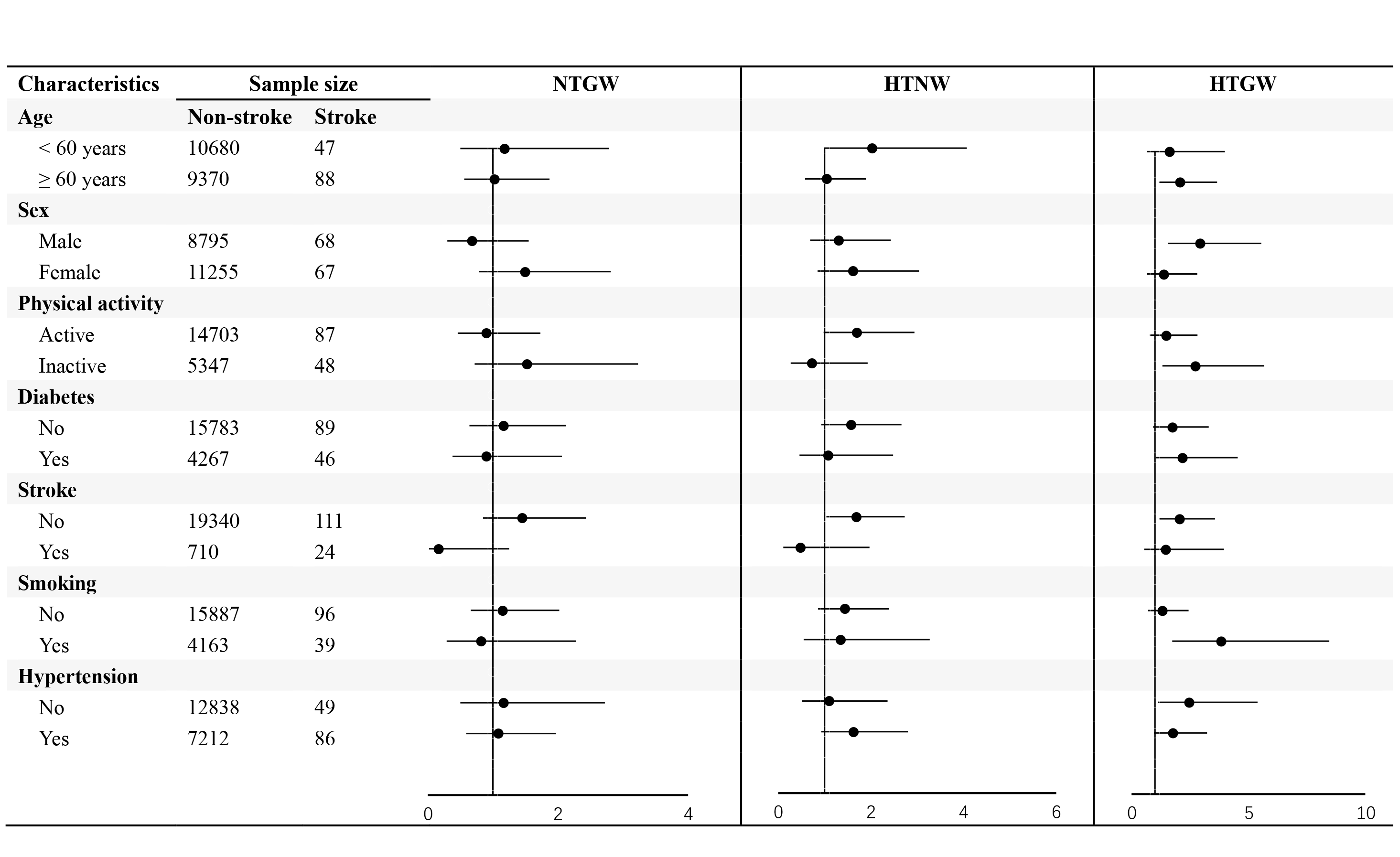

Supplement: Supplementary Figure 3 — Stratified analyses on the association between triglyceridemic-waist phenotypes and stroke risk. Adjustment for age, sex, education, smoking, alcohol drinking, physical activity, family history (hypertension, diabetes, and coronary heart disease) and medical history (hypertension, diabetes mellitus, stroke, and atrial fibrillation) except the corresponding stratification variable. [file Image_3.tif]

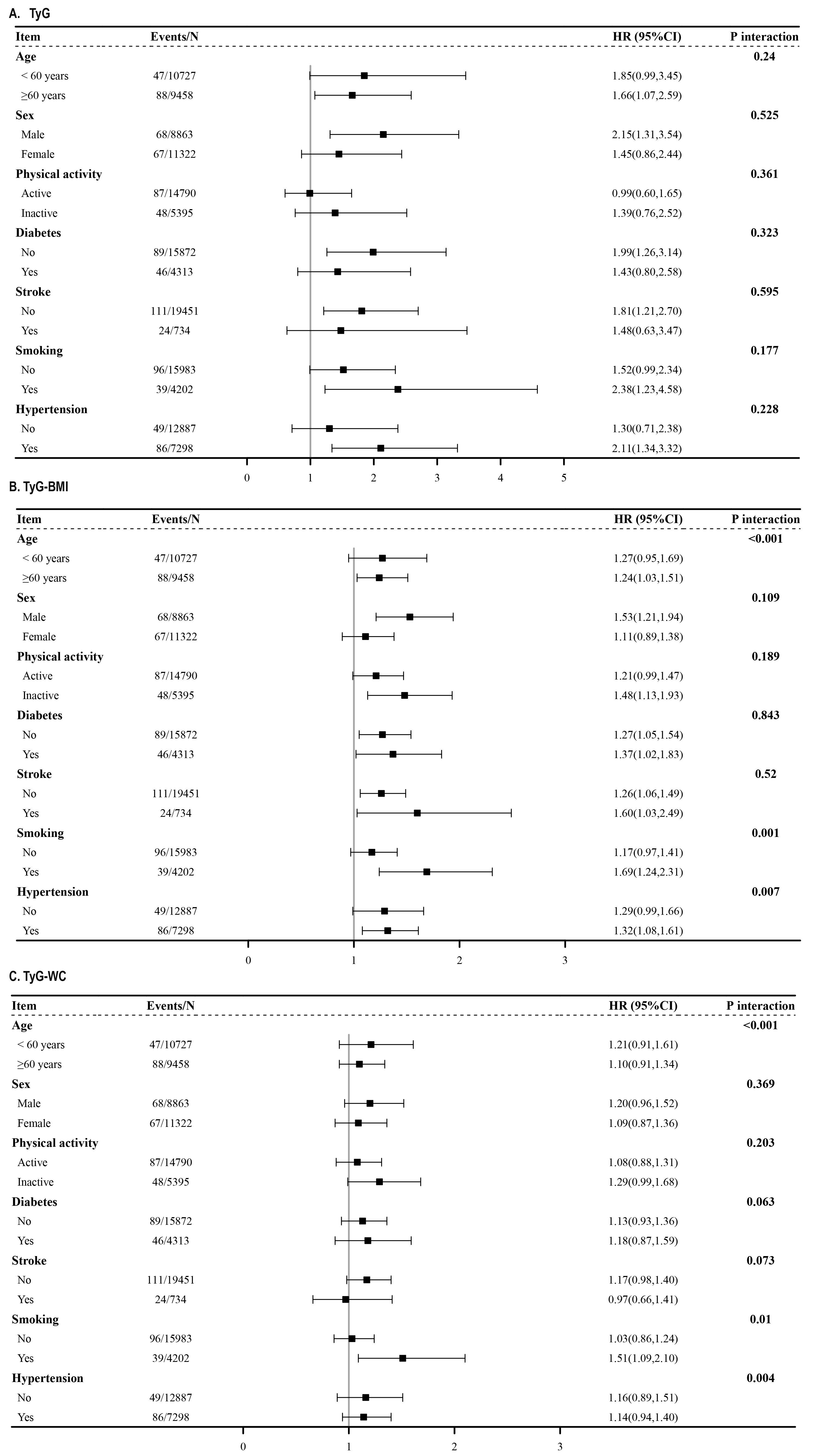

Supplement: Supplementary Figure 4 — Stratified analyses on the association between TyG, TyG-BMI, and TyG-WC and stroke risk. WC, waist circumference; BMI, body mass index; TyG, triglyceride glucose. Adjustment for age, sex, education, smoking, alcohol drinking, physical activity, family history (hypertension, diabetes, and coronary heart disease) and medical history (hypertension, diabetes mellitus, stroke, and atrial fibrillation) except the corresponding stratification variable. [file Image_4.tif]
